# Efficacy of 3D slicer-assisted minimally invasive thrombolysis combined with soft channel drainage versus craniotomy: a retrospective study in moderate basal ganglia hemorrhage

**DOI:** 10.3389/fneur.2025.1651466

**Published:** 2025-10-23

**Authors:** Huaxuan Chen, Hui Tang, Bin Shang, Bo Luo, Fei Qiao, Qiuguo He, Pengcheng Li

**Affiliations:** Department of Neurosurgery, Beijing Anzhen Nanchong Hospital of Capital Medical University, Nanchong Central Hospital, The Second Clinical Medical College of North Sichuan Medical College, Nanchong, Sichuan, China

**Keywords:** 3D slicer, basal ganglia hemorrhage, minimally invasive thrombolysis, soft channel drainage, craniotomy

## Abstract

**Background:**

The optimal clinical treatment strategy for moderate basal ganglia hemorrhage (30–50 mL) remains a subject of debate. This study aims to evaluate the clinical efficacy of 3D Slicer-assisted minimally invasive thrombolysis combined with soft channel drainage by comparing it with traditional craniotomy, thus assessing the clinical value of this minimally invasive approach.

**Methods:**

In this retrospective study, the patients were divided into the minimally invasive group and the craniotomy group based on surgical approaches. The study compared operative time, hematoma clearance rates, and postoperative complication rates between the two groups. Functional recovery was assessed at 6 months postoperatively using the modified Rankin Scale (mRS) and Barthel Index. Additionally, the study analyzed differences in medical costs and cost-effectiveness between the two treatment approaches.

**Results:**

A total of 112 patients were enrolled, with 41 in the minimally invasive group and 71 in the craniotomy group. Baseline characteristics showed no significant differences between groups (all *p* > 0.05), including demographics [e.g., sex: 30 males (73.2%) and 11 females (26.8%) vs. 51 males (71.8%) and 20 females (28.2%)], median age: [70 (63.5–76.5) years vs. 65 (58–72) years], median hematoma volume: [40.6 (37.7–47.8) mL vs. 41.2 (35.4–45.1) mL], median GCS score: [9 (8–11) vs. 10 (9–11)], median NIHSS score: [15 (11.5–18) vs. 12 (10–18)], median midline shift [3.70 (2.85–5.10) mm vs. 4.20 (3.20–5.70) mm], and proportion of intraventricular hemorrhage [6 (14.6%) vs. 14 (19.7%)]. Hematoma location and underlying diseases were also comparable. Compared to the craniotomy group, the minimally invasive group exhibited significantly less intraoperative blood loss, shorter operative time, lower rates of tracheostomy and pulmonary infection (*p* < 0.05), and smaller volume of cerebral edema at 72 h postoperatively (p < 0.05). Follow-up results 6 months postoperatively indicated that the minimally invasive group had significantly lower mRS scores and higher Barthel Index scores (*p* < 0.05). Regarding healthcare costs, expenses were lower in the minimally invasive group, and the incremental cost-effectiveness ratio (ICER) was more favorable (*p* < 0.05).

**Conclusion:**

3D Slicer-assisted minimally invasive thrombolysis combined with soft channel drainage represents an effective treatment for patients with moderate basal ganglia hemorrhage. It offers several advantages, including precise localization, minimal surgical trauma, a low complication rate, favorable safety profile, and cost-effectiveness. Therefore, this minimally invasive technique holds significant clinical value.

## 1 Introduction

Spontaneous basal ganglia hemorrhage is the primary clinical subtype of hypertensive intracerebral hemorrhage, and its incidence is closely associated with age (1). Epidemiological studies indicate that individuals aged 50 and above exhibit a higher annual incidence rate, constituting the majority of supratentorial hemorrhage cases (2). The poor prognosis of this condition is primarily attributed to two pathological mechanisms: first, the direct mechanical compression of the internal capsule corticospinal tract and thalamic nuclei by the hematoma (3); and second, the cascade of secondary cerebral edema (4). These two mechanisms act synergistically, resulting in a high in-hospital mortality rate. Even among survivors, various functional impairments, including motor, sensory, and cognitive deficits, are often observed (5). Consequently, this disease not only poses a significant threat to patients’ lives but also imposes a long-term burden of disability.

The clinical management of basal ganglia hemorrhage primarily involves two strategies: conservative treatment and surgical intervention. For large hematomas (typically >50 mL), craniotomy for hematoma evacuation is a commonly used treatment approach. However, the optimal treatment for moderate hematoma volumes (30–50 mL) remains a subject of debate. While traditional craniotomy can effectively remove hematomas, it is associated with significant drawbacks, including substantial surgical trauma, slow postoperative recovery, and a high risk of complications (6). Therefore, clinical decision-making requires a careful balance of its benefits and risks. In recent years, research has increasingly focused on minimally invasive techniques, aiming to reduce surgical trauma, minimize complications, and accelerate patient recovery. This trend reflects an ongoing pursuit of safer and more efficient treatment options.

In recent years, 3D Slicer’s precise navigation technology has demonstrated potential value in the minimally invasive treatment of basal ganglia hemorrhage. By reconstructing three-dimensional images, this technology enables accurate quantification of hematoma volume and optimization of puncture path planning, while effectively protecting the lenticulostriate artery branches (7). These functions significantly enhance the anatomical precision of the procedure, providing reliable technical support for the implementation of the sequential therapy of “local anesthesia, minimally invasive puncture, local fibrinolytic thrombolysis, and soft channel drainage.” Furthermore, the combination of phased hematoma removal and optimized drainage efficiency further reduces the risk of iatrogenic injury, thereby highlighting the clinical advantages of this technique (8). However, there remains a lack of high-quality evidence-based medical data regarding the choice of surgical approach for moderate basal ganglia hemorrhage.

This study employs a retrospective cohort design to compare the clinical outcomes of the two aforementioned surgical methods in the treatment of moderate basal ganglia hemorrhage. Accordingly, the efficacy and safety of 3D Slicer-assisted minimally invasive thrombolysis combined with soft channel drainage are evaluated.

## 2 Research subjects and methods

### 2.1 Patient selection

This study is a single-center retrospective analysis that consecutively enrolled patients with spontaneous basal ganglia hemorrhage admitted to the Department of Neurosurgery at Nanchong Central Hospital between January 2021 and December 2023. Rigorous inclusion and exclusion criteria were applied for screening, and ultimately, 112 cases meeting the study requirements were identified. The study protocol was reviewed and approved by the Ethics Committee of Nanchong Central Hospital. Based on the different surgical strategies adopted in clinical practice, patients were naturally divided into two groups: the minimally invasive group (*n =* 41), in which patients underwent 3D Slicer-assisted minimally invasive thrombolysis combined with soft channel drainage; and the craniotomy group (*n =* 71), in which patients received traditional craniotomy with hematoma evacuation. Patient grouping (i.e., surgical decision-making) was determined through comprehensive evaluation of relevant clinical factors, team discussions, and thorough doctor-patient communication to reach consensus.

The following inclusion criteria were applied in this study: (1) Diagnosis of spontaneous basal ganglia hemorrhage according to the Chinese Guidelines for the Diagnosis and Treatment of Cerebral Hemorrhage (2021), with the time from onset to the first cranial CT scan not exceeding 12 h; (2) Confirmation of basal ganglia hemorrhage (with or without intraventricular hemorrhage) by CT or MRI, with a hematoma volume of 30–50 mL; (3) Patient age between 30 and 80 years, with a Glasgow Coma Scale (GCS) score of 6–15 upon admission; (4) Absence of severe dysfunction in vital organs such as the heart, lungs, liver, or kidneys, or other systemic diseases that might interfere with treatment decisions.

Exclusion criteria were as follows: (1) Imaging showing a hematoma volume exceeding 50 mL; (2) Secondary hemorrhage (e.g., intracranial aneurysm, vascular malformation, tumor-related hemorrhage, or coagulation dysfunction); (3) Severe bleeding tendency (including a history of major bleeding within the past 3 months, uncorrected anticoagulant/antiplatelet therapy) or pregnancy/lactation; (4) Presence of confounding factors that may affect prognosis assessment (e.g., loss to follow-up, severe psychiatric history, or pre-existing disability).

All patients received standardized acute-phase management. Specific measures included hemostasis, maintenance of fluid balance, control of blood glucose and blood pressure, and implementation of stepwise intracranial pressure reduction therapy. Both groups received standardized rehabilitation services after hospital discharge.

### 2.2 Surgical methods

#### 2.2.1 Minimally invasive group

**Figure 1:**
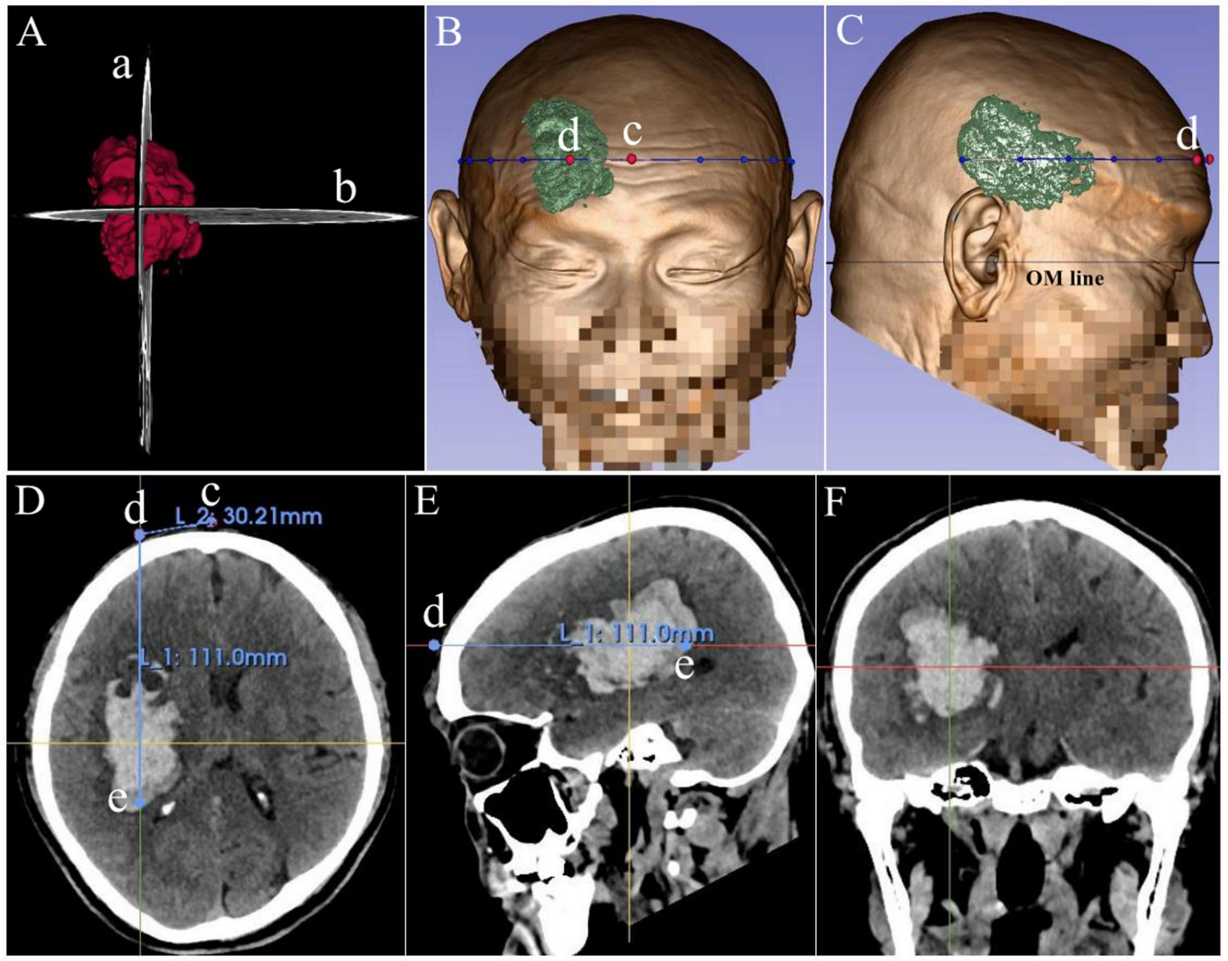
Diagram of surgical pathway planning. Panel **(A)**: 3D hematoma model; Panels **(B–C)**: 3D head reconstructions; Panels **(D–F)**: axial, sagittal, and coronal CT views. The puncture trajectory (intersection of maximal axial and sagittal planes) extends along the hematoma’s long axis. Key points: c (sagittal line–puncture plane intersection), d (scalp puncture site), e (posterior edge of maximal axial plane). Distance measurements: de (depth from puncture point to catheter tip), cd (distance from sagittal line to puncture point).

1. Planning of the minimally invasive surgical pathway (Figure 1).

The 3D Slicer software was utilized for three-dimensional reconstruction and puncture pathway planning. First, the Volume Rendering module was used to reconstruct the three-dimensional structure of the scalp and skull. Subsequently, the Segment Editor module was employed to precisely segment the intracranial hematoma to define its boundaries. The puncture pathway was designed based on the morphological characteristics of the hematoma, following its long axis. The specific location was determined by the spatial intersection line of the axial and sagittal maximal planes of the hematoma (via frontal puncture), ensuring that the tip of the drainage catheter was positioned anterior to the posterior edge of the axial maximal plane of the hematoma. Distance measurements were performed using the software’s built-in tools, calculating the linear distance between the target point and the puncture point (for intraoperative depth calibration) and the linear distance between the sagittal line of the puncture plane and the puncture point. This method enabled quantitative evaluation of hematoma spatial localization and puncture pathway planning.

1. Surgical procedure.

The patients were placed in a standard supine position. After routine skin preparation and disinfection, a sterile operative field was established. Sedation was induced by intravenous administration of propofol, followed by local infiltration anesthesia at the puncture site using 2 mL of 2% lidocaine. A 5-mm scalp incision was made at the puncture site, and a cranial drill was used to penetrate the outer table of the skull. The drill direction was adjusted to align with the sagittal and axial planes of the hematoma. Drilling continued until the inner table of the skull was penetrated. Finally, the dura mater was punctured to complete the establishment of the access channel. A 10F silicone drainage tube preloaded with a metal stylet was inserted. When the tube reached the preset depth and hematoma fluid began to flow spontaneously, the metal stylet was removed, and the tube was connected to a three-way drainage system. Intermittent negative pressure aspiration was performed using a 10 mL syringe, with the aspiration volume strictly controlled to be less than 50% of the initial hematoma volume. After the procedure, the drainage tube was sutured and fixed, and the incision was closed in layers.

1. Postoperative urokinase thrombolysis management.

**Figure 2:**
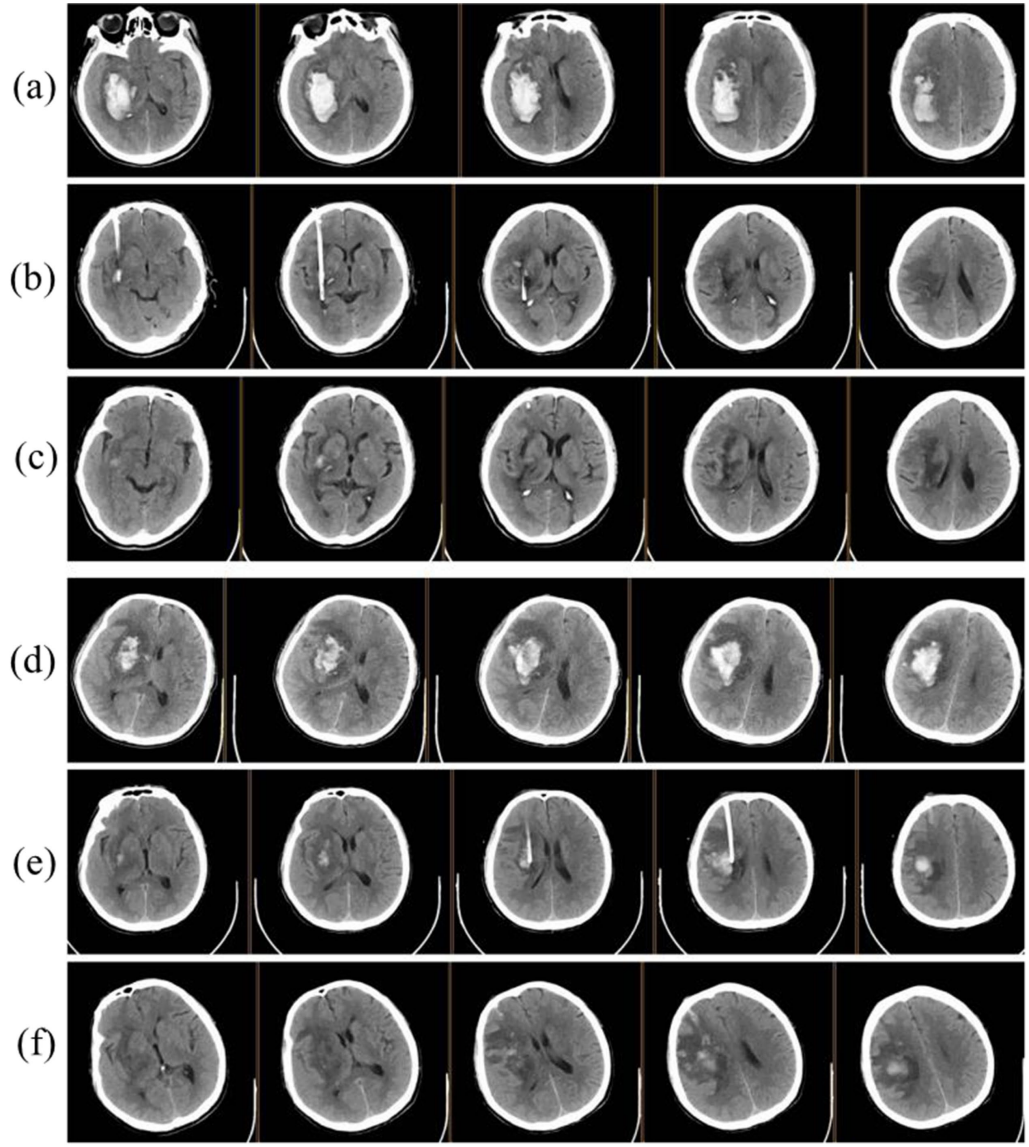
Illustration of minimally invasive surgery. **(a)** Preoperative CT showing right basal ganglia hemorrhage. **(b,c)** postoperative CT images at 24 h and 3 days. **(d)**, preoperative CT of another patient demonstrating right basal ganglia hemorrhage with significant cerebral edema. **(e,f)** postoperative CT images at 24 h and 3 days.

A CT scan was performed within 8 h postoperatively to confirm the three-dimensional position of the drainage tube, screen for rebleeding, and quantify the residual hematoma volume. If the tip of the drainage tube was located within the hematoma cavity and the residual hematoma volume exceeded 15 mL, thrombolytic therapy was initiated. A mixture of 100,000 U urokinase and 10 mL of normal saline was prepared, and 5 mL was slowly injected through the three-way valve. The tube was clamped for 2 h to allow the drug to take effect, after which the drainage was reopened. This protocol was performed twice daily, with adjustments made based on changes in the hematoma. The drainage tube was removed when the CT scan showed near-complete hematoma clearance or the drainage was deemed insufficient (Figure 2).

#### 2.2.2 Craniotomy group

The surgery was performed under general anesthesia with endotracheal intubation, utilizing an extended pterional approach to fully expose the frontotemporal region. Subsequently, an electric bone cutter was used to create a bone window with a diameter of 4 to 6 cm. After meticulous management of bone edge bleeding, the dura mater was tented to the edges of the bone window. The dura mater was then incised in a curvilinear fashion, and an operative corridor was established through the natural space of the middle temporal gyrus or lateral fissure. Under microscopic assistance, the hematoma was evacuated, and hemostasis was thoroughly achieved. Intraoperative assessment of brain tissue compliance was continuously performed; if the brain tissue tension remained high after hematoma evacuation, selective decompressive craniectomy was performed. Finally, the dura mater, temporalis muscle, and scalp were sequentially sutured to complete the procedure.

### 2.3 Observation indicators

This study employed a standardized data collection framework, with all parameters defined and measured according to international clinical research guidelines. The specific methods are as follows.

1. Baseline characteristics.

Demographic characteristics such as gender, age, smoking history, and alcohol consumption were collected. Preoperative Glasgow Coma Scale (GCS) and National Institutes of Health Stroke Scale (NIHSS) score were recorded. Imaging assessments included hematoma location, hematoma volume, midline shift distance, and intraventricular hemorrhage. Comorbidities such as hypertension, diabetes, coronary heart disease, and chronic kidney disease were also evaluated.

1. Perioperative parameters

Key perioperative time parameters, including the interval from onset to surgery and the operative time, were recorded. The first-pass success rate (defined as the proportion of cases where the target was achieved in a single puncture) and intraoperative blood loss were also documented. Hematoma clearance rates at 24 and 72 h postoperatively were calculated using the following formula: [(preoperative hematoma volume − postoperative hematoma volume) /preoperative hematoma volume] × 100%. Additionally, ICU monitoring duration and hospital stay length were collected. Postoperative 72-h cerebral edema volume was quantitatively analyzed using 3D Slicer software.

1. Postoperative complication monitoring.

Complications monitored included pulmonary infection, intracranial infection, urinary tract infection, intracranial rebleeding, cerebral infarction, stress-induced upper gastrointestinal bleeding, seizures, and tracheostomy.

1. Neurological function and prognostic assessment.

This study conducted standardized blinded evaluations for all neurological function and prognostic assessment indicators (GCS, NIHSS, Barthel Index, and mRS scores). Quantitative assessment metrics included improvements in NIHSS and GCS scores at 2 weeks postoperatively. Functional status at 6 months postoperatively was assessed using the Barthel Index and the modified Rankin Scale (mRS).

1. Health economic analysis.

Surgical-related costs and total hospitalization costs for both groups were recorded. The incremental cost-effectiveness ratio (ICER) was used to evaluate the economic differences between the two treatment strategies, calculated as: ICER = (total costs of the minimally invasive group − total costs of the craniotomy group) / (Barthel Index of the minimally invasive group − Barthel Index of the craniotomy group).

### 2.4 Statistical analysis methods

Statistical analysis was performed using SPSS 26.0 (IBM Corp.). For continuous variables, normality was assessed using the Shapiro–Wilk test (for sample sizes < 50) or the Kolmogorov–Smirnov test (for sample sizes ≥ 50). Normally distributed data were expressed as mean ± standard deviation, and intergroup comparisons were performed using independent samples *t*-tests. Non-normally distributed data were expressed as median (interquartile range), and intergroup comparisons were performed using the Mann–Whitney U test. For categorical variables, frequencies (percentages) were used for description, and intergroup comparisons were performed using the chi-square test (for expected frequencies ≥ 5) or Fisher’s exact test (for expected frequencies < 5). All hypothesis tests were two-tailed, with a significance level set at *p* < 0.05.

## 3 Results

### 3.1 Comparison of baseline characteristics

A total of 112 patients were enrolled in this study, with 41 assigned to the minimally invasive group and 71 to the craniotomy group. No significant differences were observed in baseline characteristics between the two groups (all *p* > 0.05). The minimally invasive group consisted of 30 males (73.2%) and 11 females (26.8%), with a median age of 70 years (IQR 63.5–76.5), while the craniotomy group included 51 males (71.8%) and 20 females (28.2%), with a median age of 65 years (IQR 58–72). Key hematoma and neurological variables were also comparable between the two groups: preoperative hematoma volume [40.6 (37.7–47.8) mL vs. 41.2 (35.4–45.1) mL], preoperative GCS score [9 (8–11) vs. 10 (9–11)], preoperative NIHSS score [15 (11.5–18) vs. 12 (10–18)], midline shift [3.70 (2.85–5.10) mm vs. 4.20 (3.20–5.70) mm], and the proportion of patients with intraventricular hemorrhage [6 (14.6%) vs. 14 (19.7%)]. Additionally, no statistically significant differences were found in terms of hematoma location or underlying diseases. Regarding the treatment approach, a difference was noted in anesthesia method: the craniotomy group exclusively received general anesthesia, whereas the minimally invasive group underwent local anesthesia. Detailed data are presented in Table 1.

**Table 1:** Comparison of baseline characteristics between the two groups.

Variable	Minimally invasive group (*n =* 41)	Craniotomy group (*n =* 71)	*p-*value
Gender			0.879
Male, *n* (%)	30 (73.2)	51 (71.8)	
Female, *n* (%)	11 (26.8)	20 (28.2)	
Age (years), median (IQR)	70 (63.5–76.5)	65 (58–72)	0.061
Smoking history, *n* (%)	19 (46.3)	28 (39.4)	0.476
Alcohol consumption history, *n* (%)	23 (56.1)	31 (43.7)	0.205
Preoperative Hematoma volume (mL), median (IQR)	40.6 (37.7–47.8)	41.2 (35.4–45.1)	0.222
Hypertension, *n* (%)	38 (92.7)	65 (91.5)	1.000
Diabetes, *n* (%)	6 (14.6)	8 (11.3)	0.604
Coronary disease, *n* (%)	3 (7.3)	5 (7.0)	1.000
Chronic kidney disease, *n* (%)	4 (9.8)	5 (7.0)	0.882
Midline shift (mm), median (IQR)	3.70 (2.85–5.10)	4.20 (3.20–5.70)	0.069
Hematoma location			0.307
Left side, *n* (%)	19 (46.3)	40 (56.3)	
Right side, *n* (%)	22 (53.7)	31 (43.7)	
Intraventricular hemorrhage, *n* (%)	6 (14.6)	14 (19.7)	0.499
Preoperative GCS score, median (IQR)	9 (8–11)	10 (9–11)	0.348
Preoperative NIHSS score, median (IQR)	15 (11.5–18)	12 (10–18)	0.069
Type of anesthesia			<0.001
Local anesthesia, *n* (%)	41 (100.0)	0 (0.0)	
General anesthesia, *n* (%)	0 (0.0)	71 (100.0)	

IQR, Interquartile range.

### 3.2 Comparison of perioperative outcomes

The minimally invasive group demonstrated significant advantages in several perioperative metrics. First, the accuracy of catheter placement was evaluated using postoperative cranial CT scans, which demonstrated a 100% first-attempt success rate of puncture in the minimally invasive group. Second, the operative time was significantly shorter in the minimally invasive group [24 (20.5–25) minutes vs. 180 (135–185) minutes], with notably less intraoperative blood loss [3 (2–3) mL vs. 178 (168–200) mL] (both *p* < 0.001). Additionally, the minimally invasive group exhibited smaller cerebral edema volumes [7.6 (5.6–8.6) mL vs. 14.8 (12.4–16.7) mL] at 72 h postoperatively (*p* < 0.001). Regarding recovery, the minimally invasive group had shorter ICU monitoring stays [3 (2–4) days vs. 7 (5–8) days] and hospital stays [(19.98 ± 9.85) days vs. (27.41 ± 9.26) days] compared to the craniotomy group (both p < 0.001). Although the hematoma clearance rate at 24 h postoperatively was lower in the minimally invasive group [70.07 (67.27–72.45)% vs. 92.52 (86.58–94.84)%], no significant difference was observed between the two groups at 72 h postoperatively [89.70 (85.76–91.91)% vs. 92.59 (86.58–94.94)%] (Table 2).

**Table 2:** Comparison of perioperative outcomes.

Variable	Minimally invasive group (*n =* 41)	Craniotomy group (*n =* 71)	*p-*value
Onset-to-surgery interval			0.631
≥24 h, *n* (%)	5 (12.2)	11 (15.5)	
<24 h, *n* (%)	36 (87.8)	60 (84.5)	
Operative time (mins), median (IQR)	24 (20.5–25)	180 (135–185)	<0.001
Blood loss (mL), median (IQR)	3 (2–3)	178 (168–200)	<0.001
1 day postoperative hematoma evacuation rate, median (IQR), %	70.07 (67.27–72.45)	92.52 (86.58–94.84)	<0.001
3 days postoperative hematoma evacuation rate, median (IQR), %	89.70 (85.76–91.91)	92.59 (86.58–94.94)	0.070
Hospital stay (days), mean ± SD	19.98 ± 9.85	27.41 ± 9.26	<0.001
ICU monitoring duration (days), median (IQR)	3 (2–4)	7 (5–8)	<0.001
Surgery-related costs, median (IQR), kCNY	7.2 (5.9–8.3)	28.4 (24.4–30.7)	<0.001
Hospitalization costs, median (IQR), kCNY	53.9 (32.4–75.1)	87.9 (77.8–103.1)	<0.001
Postoperative cerebral edema volume (72 h), median (IQR), mL	7.6 (5.6–8.6)	14.8 (12.4–16.7)	<0.001

IQR, Interquartile range; kCNY, kilo Chinese Yuan.

### 3.3 Comparison of postoperative complications

The minimally invasive group had significantly lower rates of tracheostomy (7.3% vs. 22.5%) and pulmonary infection (19.5% vs. 38.0%) compared to the craniotomy group (both *p* < 0.05). Regarding postoperative intracranial rebleeding, one case (2.4%) occurred in the minimally invasive group, while three cases (4.2%) occurred in the craniotomy group, with no statistically significant difference. All rebleeding cases involved minor hemorrhages that resolved spontaneously without additional surgical intervention. The craniotomy group showed slightly higher rates of cerebral infarction (4.2% vs. 0%) and intracranial infection (2.8% vs. 0%), though these differences were not statistically significant. Furthermore, the two groups had similar incidences of gastrointestinal bleeding, seizures, and urinary tract infections (Table 3).

**Table 3:** Comparison of postoperative complications between the two groups.

Variable	Minimally invasive group (*n =* 41)	Craniotomy group (*n =* 71)	*p-*value
Intracranial infection, *n* (%)	0 (0)	2 (2.8)	0.532
Urinary tract infection, *n* (%)	2 (4.9)	3 (4.2)	1.000
Postoperative pulmonary infection, *n* (%)	8 (19.5)	27 (38.0)	0.042
Tracheotomy, *n* (%)	3 (7.3)	16 (22.5)	0.039
Post-operative rebleeding, *n* (%)	1 (2.4)	3 (4.2)	1.000
Postoperative cerebral infarction, *n* (%)	0 (0)	3 (4.2)	0.467
Epileptic seizures, *n* (%)	1 (2.4)	2 (2.8)	1.000
Stress-induced upper gastrointestinal bleeding, *n* (%)	8 (19.5)	21 (29.6)	0.241

### 3.4 Neurological function and prognostic evaluation

At 2 weeks postoperatively, both groups showed significant improvements in GCS and NIHSS scores compared to preoperative assessments (*p* < 0.05). The minimally invasive group achieved significantly higher GCS scores [13 (9.5–14) points vs. 11 (9–12) points] and notably lower NIHSS scores [6 (4–12) points vs. 9 (7–11) points] than the craniotomy group (*p* < 0.05). At the 6–month follow–up, the minimally invasive group exhibited significantly lower mRS scores [3 (2–3) points vs. 3 (2–4) points] and significantly higher Barthel Index values [75 (70–90) points vs. 70 (45–85) points] (*p* < 0.05) (Table 4).

**Table 4:** Comparison of neurological function and daily living ability between the two groups.

Variable	Minimally invasive group (*n =* 41)	Craniotomy group (*n =* 71)	*p-*value
Pre-surgery			
GCS score, median (IQR)	9 (8–11)	10 (9–11)	0.348
NIHSS score, median (IQR)	15 (11.5–18)	12 (10–18)	0.069
2 weeks post-surgery			
GCS score, median (IQR)	13 (9.5–14)*	11 (9–12)^*^	0.007
NIHSS score, median (IQR)	6 (4–12)#	9 (7–11)^#^	0.004
6 months post-surgery			
mRS score, median (IQR)	3 (2–3)	3 (2–4)	0.017
Barthel index, median (IQR)	75 (70–90)	70 (45–85)	0.011

IQR, Interquartile range. Compared to preoperative GCS score, *P < 0.05; Compared to preoperative NIHSS score, ^#^*P* < 0.05.

### 3.5 Health economic evaluation

The surgery-related costs [7.2 (5.9–8.3) kCNY vs. 28.4 (24.4–30.7) kCNY] and hospitalization costs [53.9 (32.4–75.1) kCNY vs. 87.9 (77.8–103.1) kCNY] were significantly lower in the minimally invasive group (*p* < 0.001) (Table 2). Furthermore, the minimally invasive group demonstrated an incremental cost-effectiveness ratio (ICER) of −6,800 CNY per Barthel index point. This indicates that compared to the craniotomy group, the minimally invasive approach reduced medical costs by 6,800 CNY for each 1-point improvement in Barthel index score.

## 4 Discussion

### 4.1 Pathological mechanisms of basal ganglia hemorrhage and innovations in minimally invasive treatment

The pathological mechanisms of basal ganglia hemorrhage primarily involve two processes. First, the mass effect of the hematoma causes direct mechanical compression. Second, toxic substances (such as heme and iron ions) released during erythrocyte lysis trigger oxidative stress reactions. These mechanisms act synergistically, disrupting the blood–brain barrier, exacerbating cerebral edema, and inducing neuronal apoptosis (9). Although traditional craniotomy can rapidly evacuate the hematoma, the procedure requires retraction of brain tissue and disruption of cortical vessels, potentially leading to secondary injury (10). This limitation helps explain the findings of the STICH trials, which demonstrated no significant improvement in patient outcomes following open surgery (11, 12).

Advances in precision medicine have driven the development of minimally invasive treatments for basal ganglia hemorrhage. Among these, minimally invasive thrombolysis combined with soft-channel drainage has shown notable advantages. This technique employs a staged hematoma evacuation strategy (with continuous postoperative drainage for 1–3 days) and shows improvement in three key areas. First, the precision of puncture trajectories has significantly improved. Using 3D Slicer reconstruction technology, surgeons can clearly visualize the course of the middle meningeal artery. A transfrontal approach effectively protects the lenticulostriate arteries, reducing the risk of puncture-related hemorrhage (13). Additionally, this approach avoids the posterior limb of the internal capsule and thalamic radiation, minimizing postoperative motor dysfunction (14). Second, surgical instruments and procedural workflows have been optimized. The use of a 4-mm flexible silicone drainage tube minimizes brain tissue damage (15). Catheter placement along the hematoma’s long axis expands the coverage of urokinase, thereby reducing residual hematoma rates (16). Postoperatively, the catheter position can be adjusted as needed to optimize hematoma clearance. Third, this technique enables molecular-level detoxification. Urokinase exerts a dual effect by activating the plasminogen-plasmin system: it dissolves clots while promoting the metabolism of harmful substances. This mechanism effectively suppresses oxidative stress (17). When combined with dynamic drainage, it further enhances the clearance efficiency of toxic compounds.

The key surgical parameters in this study drew upon evidence from influential international trials. Specifically, the urokinase thrombolysis trigger threshold (>15 mL residual hematoma volume) directly reflected the MISTIE III trial’s pivotal finding: final hematoma volumes ≤15 mL significantly improved functional outcomes (mRS scores 0–3) (18). Consequently, we implemented 15 mL as the thrombolysis initiation standard to optimize hematoma clearance. For the ≤50% single-aspiration volume limit, we considered two critical factors: safety (excess aspiration increases intraoperative rebleeding or mechanical tissue injury risks), and strategy (adopting MISTIE III’s 72-h phased clearance approach with 8-h thrombolytic dosing, which balances efficacy against procedural risks). By combining this aspiration limit with staged operations, our protocol strictly followed this graduated clearance principle to maximize safety.

Although neuronavigation systems can precisely localize intracranial hematomas, their complexity (requiring 45–60 min on average) and high cost (often ranging from hundreds of thousands to millions of CNY) limit their utility in emergency settings (19). In contrast, 3D Slicer-based navigation platforms offer distinct advantages. These open-source systems can operate on standard CT workstations, with a simplified workflow that completes 3D reconstruction and surgical planning within 15 min. Clinical studies demonstrate that this technology significantly reduces preoperative decision-making time in primary care hospitals while effectively extending the therapeutic time window (20). This cost-effective solution provides a new option for emergency neurosurgical care.

### 4.2 Neuroprotective mechanisms of minimally invasive intervention

The results of this study demonstrate that the minimally invasive group exhibited shorter operative time, less intraoperative blood loss, and reduced hospitalization stay. These findings align with previous research by Yan et al. and Luan et al., further confirming the advantages of minimally invasive techniques in improving surgical efficiency (21, 22). From a pathophysiological perspective, the two surgical approaches differ significantly in their impact on brain tissue. Traditional craniotomy requires the creation of a larger bone window and brain retraction, which may activate the MMP-9 signaling pathway, thereby disrupting the blood–brain barrier and triggering vasogenic edema (23). Imaging data revealed that the minimally invasive group had significantly smaller cerebral edema volumes than the craniotomy group at 72 h postoperatively. In this study, preoperative trajectory planning was performed using 3D Slicer software, enabling precise targeting of the puncture site and optimized control of the aspiration range, thereby effectively reducing mechanical damage to normal brain tissue.

Regarding hematoma evacuation efficiency, no significant difference was observed between the two groups at 3 days postoperatively (minimally invasive group: 89.70% vs. craniotomy group: 92.59%). However, the minimally invasive group demonstrated better outcomes in mitigating cerebral edema. This finding suggests that the speed of hematoma evacuation may not be the decisive factor in improving prognosis, whereas the extent of mechanical injury during surgery may play a more critical role. These results support the “minimal trauma threshold” theory, which posits that surgical intervention must balance evacuation efficiency with procedural trauma (24).

Functional outcomes further demonstrated the superiority of minimally invasive surgery. At the 6-month follow-up, the minimally invasive group showed lower mRS scores than the craniotomy group. These findings align with authoritative international evidence, particularly the ENRICH trial which—while focused specifically on lobar hemorrhage patients—similarly reported better 180-day mRS performance with minimally invasive techniques (24). Additionally, Barthel Index assessments revealed enhanced recovery of activities of daily living in the minimally invasive group. This finding is corroborated by Li et al., who reported significantly higher Barthel Index scores in patients undergoing minimally invasive surgery (25). These benefits may stem from the technical features of minimally invasive procedures, such as the use of a 4-mm drainage tube to establish the operative channel and limiting cerebrospinal fluid loss to <10 mL.

In summary, the neuroprotective effects of minimally invasive surgery are primarily reflected in the following aspects: (1) reducing mechanical damage to normal brain tissue through precise preoperative planning and minimally invasive techniques; (2) effectively mitigating blood–brain barrier disruption and vasogenic edema; and (3) optimizing hematoma evacuation while minimizing surgical trauma, thereby improving neurological functional outcomes.

### 4.3 Anesthesia modality and complication risk in patients with basal ganglia hemorrhage

Patients with basal ganglia hemorrhage often present with underlying conditions such as hypertension and diabetes, which may compromise their tolerance to general anesthesia. This study demonstrates that local anesthesia for minimally invasive surgery offers significant advantages over general anesthesia for craniotomy.

Notably, anesthesia method and surgical type showed perfect collinearity. All minimally invasive procedures used local anesthesia, while all craniotomies used general anesthesia. This made traditional multivariable analysis unsuitable for separating their independent effects. Following principles of managing confounding factors, we analyzed the effects separately based on clinical mechanisms.

Regarding complication control, the first key factor was the independent effect of anesthesia: local anesthesia independently benefits patients by avoiding respiratory and circulatory depression caused by general anesthetics. Our results show significantly lower rates of postoperative pneumonia and tracheostomy in the minimally invasive group versus the craniotomy group. This aligns with findings by Guan et al. (26). The key advantage is preserving the natural cough reflex. This reduces aspiration risk, consistent with studies by Gong et al. (27) and Mao et al. (28) confirming general anesthesia elevates pneumonia risk. The second factor was the independent effect of surgical approach: the minimally invasive technique itself independently reduces complications by minimizing surgical trauma. Shorter operation times and less blood loss improve tissue damage control. Furthermore, these factors work synergistically: Local anesthesia enhances perioperative safety by reducing physiological suppression, while minimally invasive surgery optimizes recovery by limiting tissue disruption. From a health economics perspective, the minimally invasive approach offers dual advantages. It shortens preoperative preparation time and reduces reliance on complex anesthesia equipment (29).

In summary, combining local anesthesia with minimally invasive surgery reduces complication risks through independent and synergistic effects. It also significantly lowers medical costs. For basal ganglia hemorrhage patients with comorbidities, this integrated strategy represents a potentially optimal treatment choice.

### 4.4 Health economic value analysis

The cost data in this study were directly extracted from the hospital information system. Results showed that the total medical costs were significantly lower in the minimally invasive group than in the craniotomy group. This difference primarily stemmed from two factors: shorter intensive care unit stays and lower surgical expenses in the minimally invasive group. While pivotal clinical trials such as MISTIE III and ENRICH have established the clinical value of minimally invasive techniques, they did not provide economic comparisons. Our cost findings thus address this evidence gap. The cost advantages align with Yang et al.’s (30) and Xu et al.’s (31) conclusions in similar populations, collectively supporting the cost-effectiveness of minimally invasive techniques.

For outcome assessment, we selected the Barthel Index as the effectiveness measure due to its direct reflection of functional independence. Using the standard ICER formula [(C_m − C_o)/(E_m − E_o)], we obtained a value of −6,800 CNY per Barthel Index point. This result indicates that minimally invasive techniques not only reduce costs but also improve functional outcomes, consistent with previous research (32). Although no predefined cost-effectiveness threshold was set, post-hoc analysis showed the results fell within WHO’s recommended acceptable range (1–3 times GDP per capita per QALY).

Limitations should be acknowledged: indirect costs (e.g., productivity losses, long-term care) and extended rehabilitation expenses were excluded. Given the higher post-craniotomy disability rates, long-term care costs may further increase its economic burden. Overall, this evidence supports minimally invasive techniques as effective solutions for improving clinical outcomes while reducing direct economic burdens on healthcare systems.

### 4.5 Limitations

This study has several limitations. First, the relatively small sample sizes in this study (minimally invasive group: *n =* 41; craniotomy group: *n =* 71) may impact reliability for some statistical inferences, but key findings remain robust. Adequate power for primary outcomes: Significantly different metrics (operative time, blood loss, hospitalization duration, costs, functional scores) showed moderate-to-large effect sizes (Cohen’s d = 0.6–4.2; RR = 0.32–1.5) with post-hoc power >76% (*α* = 0.05), strengthening core conclusions. Detection limitations for secondary metrics: Statistical power was inadequate (<30%) for small-effect outcomes (re-bleeding) and low-frequency events (cerebral infarction), suggesting possible Type II errors. Consequently, larger cohorts are recommended to enhance statistical power in future studies. Second, as a single-center retrospective analysis conducted in China, this study’s cost-effectiveness conclusions may be influenced by specific healthcare settings and insurance policies. We recommend subsequent multinational, multicenter validation studies following the TREND statement guidelines to evaluate the generalizability of these findings across different healthcare systems. In addition, the 6-month follow-up period in this study may be insufficient to fully evaluate patients’ long-term functional recovery. Future studies should consider extending the follow-up duration and collecting quality-of-life data according to PRO (Patient-Reported Outcomes) standards to obtain more comprehensive outcome assessments. Finally, long-term rehabilitation expenses, household care burdens, and community rehabilitation resource consumption were excluded as indirect costs. To address these gaps, we recommend using ISPOR standards in future research. Combining EQ-5D instruments with household care diaries would enable comprehensive full-cycle cost-effectiveness evaluations.

## 5 Conclusion

The 3D Slicer-assisted minimally invasive thrombolysis combined with soft-channel drainage achieves high efficacy-safety profiles for moderate basal ganglia hemorrhage. Compared with traditional craniotomy, this approach not only effectively evacuates hematomas but also reduces trauma and postoperative complications. Moreover, the minimally invasive procedure incurs lower medical costs, exhibits higher cost-effectiveness, and contributes to optimized healthcare resource allocation. This approach is particularly suitable for implementation in medically underserved regions due to its minimal equipment requirements. Given these advantages, this technique holds significant clinical value, particularly for high-risk patients or those with limited tolerance to invasive procedures.

## Data Availability

The original contributions presented in the study are included in the article/supplementary material, further inquiries can be directed to the corresponding author.
